# Spontaneus Suburothelial Hemorrhage Associated With Forniceal Rupture and Retroperitoneal Hemorrhage

**DOI:** 10.7759/cureus.23728

**Published:** 2022-04-01

**Authors:** Rainel Zelaya, Douglas Byerly, Anthony Zarka

**Affiliations:** 1 Radiology, San Antonio Uniformed Services Health Education Consortium, San Antonio, USA; 2 Radiology, Uniformed Services University of the Health Sciences, Bethesda, USA; 3 Radiology, Wilford Hall Ambulatory Surgical Center, San Antonio, USA; 4 Pediatric Radiology, Baylor College of Medicine, San Antonio, USA; 5 Radiology, Children’s Hospital of San Antonio, San Antonio, USA

**Keywords:** bleeding diathesis, gross hematuria, hematuria, therapeutic anticoagulation, spontaneous retroperitoneal hemorrhage, forniceal rupture, suburothelial hemorrhage

## Abstract

Spontaneous suburothelial hemorrhage is a rare process of unclear pathologic etiology, although it has been associated with bleeding diathesis and anticoagulation. The most common clinical presentation is acute onset flank pain and macroscopic hematuria. On imaging, there is a mural thickening of the renal pelvis and upper ureters leading to luminal narrowing. Despite luminal narrowing, hydronephrosis has only rarely been reported in the association. The imaging finding of mural thickening has led to the misdiagnosis as malignancy, resulting in unnecessary nephrectomy. Suburothelial hemorrhage can be unilateral or bilateral, although the majority of reported cases are unilateral. We present a case of a patient with bilateral spontaneous suburothelial hemorrhage with forniceal rupture and spontaneous retroperitoneal hemorrhage, a unique triad compared to prior cases presented in the literature.

## Introduction

Spontaneous suburothelial hemorrhage is a rare pathologic process most commonly associated with anticoagulation, although other causes of bleeding diathesis such as hemophilia have been documented [1]. Patients present with acute onset flank pain and macroscopic hematuria, nonspecific features which are more commonly associated with nephrolithiasis, urothelial malignancy, or infection. Other associations which have been included in the literature include trauma, hypertension, diabetes, drug abuse, and amyloidosis. Historically, misdiagnosis of suburothelial hemorrhage as malignancy has led to numerous cases of unnecessary nephrectomy [1-3]. This is likely due to the similarities in clinical and imaging presentation, in addition to inexperience or lack of knowledge regarding suburothelial hemorrhage. Our case represents a unique triad of suburothelial hemorrhage, forniceal rupture, and retroperitoneal hemorrhage. We will briefly discuss the imaging features of our case and clues to differentiate suburothelial hemorrhage from malignancy.

## Case presentation

The patient was an African American 72-year-old female with a history of chronic warfarin therapy for a combined history of deep venous thromboses of the lower extremities, strokes, and atrial fibrillation. She was found to have an international normalized ratio (INR) of 18 (normal: 2.0-3.0) by her primary care provider, for which she was immediately sent to the emergency room. The patient reported no recent history of trauma and complained of some vague symptoms, most notable for lower abdominal and bilateral leg pain and macroscopic hematuria. She denied fever, chills, nausea, vomiting, dysuria, and dizziness, but complained of some generalized fatigue. At presentation, the patient demonstrated mild tachycardia with a heart rate of 106 beats per minute, systolic blood pressure of 113 mmHg, and diastolic blood pressure of 65 mmHg. The patient was otherwise afebrile with a normal respiratory rate and oxygen saturation. Physical examination of the patient revealed a nondistended abdomen without significant tenderness. There was no evidence of costovertebral angle tenderness. There was no evidence of guarding, rebound, or palpable/pulsatile mass. Despite the benign abdominal exam, the available clinical and laboratory findings at the time remained concerning for a source of bleeding, which prompted the emergency physician to order a CT scan. Laboratory and diagnostic tests obtained later in the emergency department revealed a decreased hemoglobin level at 6.5 grams per deciliter (g/dL), below the patient’s baseline ranging between 8.5 and 9 g/dL (normal: 11.6-15.0 g/dL). The patient’s lactate was also mildly elevated at 3.5 mmol/L (normal: 0.5-1.0 mmol/L). The patient was given an intravenous bolus of normal saline and a blood transfusion was later initiated. The patient was admitted with conservative management, including further fluid resuscitation and discontinuation of her anticoagulation with correction of her INR. She was discharged in stable condition with a resolution of her symptoms and scheduled for a follow-up with her primary care doctor to consider the risk versus benefit discussion of continuing anticoagulation.

**Figure 1:**
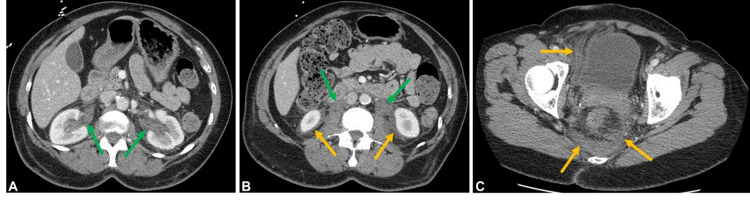
Axial contrast-enhanced CT of the abdomen and pelvis in the portal venous phase through the interpolar kidneys (A), lower pole kidneys (B), and through the bladder and pelvis (C) demonstrates marked and hyperdense circumferential thickening of both renal pelves extending to the ureters (green arrows). There is also hyperdense fluid consistent with hemorrhage in the bilateral perinephric regions and retroperitoneum, tracking down around the bladder and in the presacral region within the pelvis (orange arrows). The abdominal aorta appears intact.

**Figure 2:**
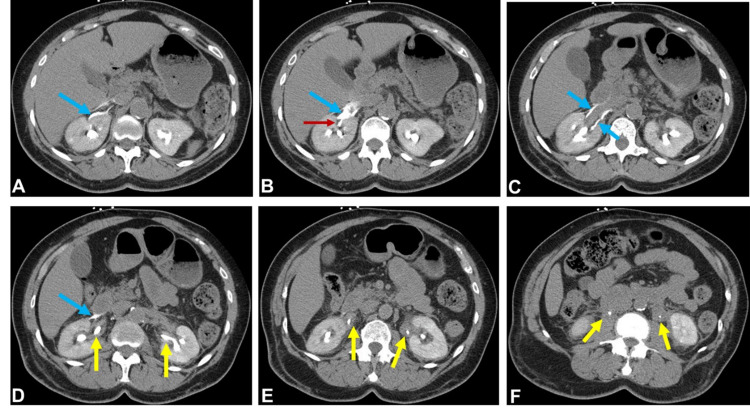
Axial contrast-enhanced CT of the abdomen obtained in the renal excretory phase with consecutive images through the kidneys (A-D) demonstrate accumulation of contrast outside of the collecting system and in the right perirenal space, also outlining the right renal vein (blue arrows). A focus of contrast contributing to this collection was seen arising from a right superior pole fornix (red arrow), compatible with forniceal rupture. Images obtained through the bilateral renal pelves (D), proximal ureters (E) and more caudal ureters (F) demonstrate prominent and high-density circumferential thickening of both renal pelves and ureters (yellow arrows).

**Figure 3:**
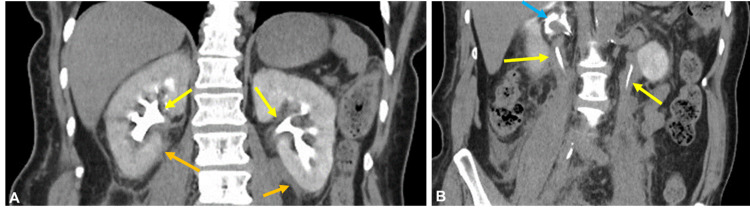
Reconstructed coronal contrast-enhanced CT images of the abdomen and pelvis obtained in the renal excretory phase with consecutive images through the kidneys (A) and ureters (B) again demonstrate the hyperdense fluid likely consisting of mixed hemorrhagic products and urine (orange arrows), with mural thickening involving the renal pelves bilaterally (yellow arrows). There is a lack of dilation of the collecting system despite narrowed lumen of the collecting system. Extravasated retroperitoneal contrast is again seen surrounding the right renal vein (blue arrow).

**Figure 4:**
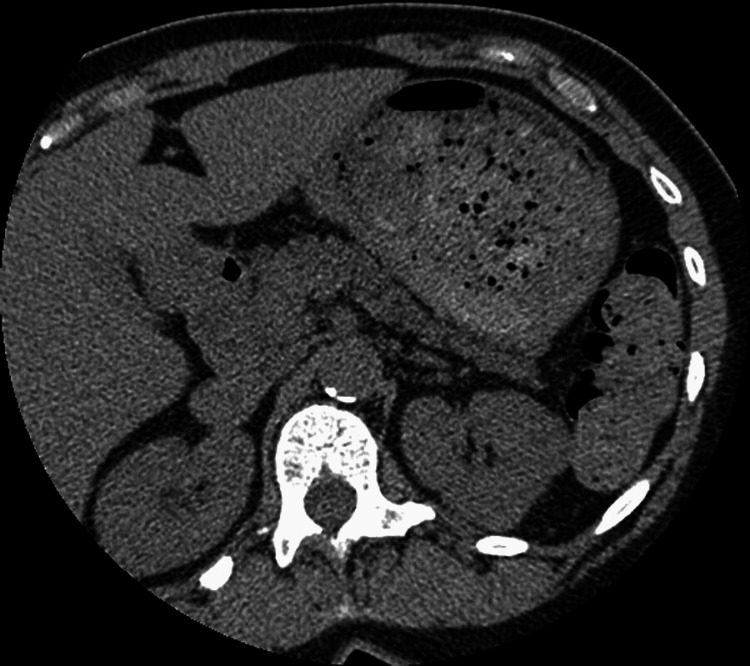
Cardiac CT examination from a year prior demonstrating a normal non-contrast appearance of the kidneys and retroperitoneum.

An abdominal CT with intravenous contrast was performed in the portal venous phase, which revealed hyperdense fluid (Hounsfield units >30) of the bilateral perinephric regions extending along both sides of the retroperitoneum, with some fluid tracking down to the pelvis concerning retroperitoneal hemorrhage (Figures 1A-1C). No urinary stones were appreciated. There was a high density, circumferential mural thickening of the bilateral renal pelves. Delayed imaging in the renal excretory phase was obtained, which revealed extravasation of contrast from a right upper pole renal fornix into the right perinephric and retroperitoneal regions, compatible with forniceal rupture (Figures 2A-2F, 3A, 3B). Redemonstration of bilateral high-density circumferential suburothelial wall thickening of both renal pelves and ureters resulting in narrowing of the bilateral renal collecting systems without evidence of obstruction (i.e., hydronephrosis or hydroureter) (Figures 2A-2F, 3A, 3B). No other sources of contrast extravasation were seen in either portal venous or delayed imaging. The abdominal aorta demonstrated atherosclerotic calcification, but otherwise appeared intact. A comparison cardiac CT from a year prior demonstrated normal noncontrast appearance of the visualized kidneys and retroperitoneum (Figure 4).

## Discussion

Abdominal pain with hematuria is a common cause of presentation to the emergency department leading to CT evaluation. Hematuria and abdominal pain are most commonly related to urinary tract infections, or stones, with malignancy a less common but more serious etiology. Suburothelial hemorrhage is a rare, benign, reversible process with a similar clinical presentation to the previously mentioned processes and a similar imaging appearance to malignancy [1-4]. In fact, suburothelial hemorrhage was initially described in 1948 by Antopol and Goldman, who presented seven patients with hematuria and renal collecting system filling defects suspicious for malignancy leading to nephrectomy but found to have only suburothelial hemorrhage [2]. Subsequently, there have been numerous additional case presentations of suburothelial hemorrhage masquerading as a transitional cell neoplasm leading to nephrectomy [1-4]. This suggests unfamiliarity of the process both by the clinicians assessing the patient and the radiologists reviewing the images leading to invasive and unnecessary treatment/surgery.

Understanding the imaging features of suburothelial hemorrhage in various phases of CT imaging is essential to establishing the correct diagnosis. Unlike other etiologies of mural thickening or compromise of the lumen of the renal collecting system, suburothelial hemorrhage tends to be hyperdense on unenhanced CT with a more diffuse circumferential involvement [1,5-8]. On the nephrographic and portal venous phases, the hyperdense mural thickening may be less conspicuous and/or mistaken for infection secondary to urothelial enhancement [1,5-8]. Delayed (excretory) phase imaging will demonstrate the mural thickening and luminal narrowing but mask the hyperdense appearance of hemorrhage [1,5-8]. Despite the luminal narrowing, hydronephrosis is an uncommon feature reported in the literature and when present is only mild [1]. Although hydronephrosis and hydroureter were absent in our case, there was a right-sided forniceal rupture suggesting the presence of increased renal pelvis pressure. In addition, most cases previously presented in the literature were unilateral with only three cases of bilateral suburothelial hemorrhage [1]. Bilateral involvement is more suggestive of a systemic process than malignancy. When suburothelial hemorrhage is suspected the patient's medical records should be reviewed for anticoagulation therapy or other bleeding diatheses such as hemophilia or factor V deficiency [1,9,10]. Other associations reported in the literature include trauma, diabetes, hypertension, drug abuse, and amyloidosis [1]. Review of the reported cases in the literature indicates each kidney is equally affected and no sex predilection [1]. While spontaneous hemorrhage of other organs, epistaxis, oral mucosal bleeding, or petechiae have been reported and aid in the diagnosis, these findings have only rarely been reported and were not present in our case.

Once the appropriate diagnosis of suburothelial hemorrhage is established, treatment involves reversal of anticoagulation and/or treatment of the cause of coagulopathy [1, 11-13]. Additional treatments such us intravenous fluids and/or blood transfusions can be considered if clinically indicated to treat symptomatic hypotension/hypovolemia and/or anemia. When follow-up imaging has been performed, the hemorrhage has been shown to resolve in one to two weeks. There are currently no follow-up imaging guidelines in the literature, although follow-up is of little clinical value if the patient's symptoms have resolved and in the absence of any features that would be worrisome for an underlying malignant process.

## Conclusions

Suburothelial hemorrhage is a rare process which can lead to the common clinical presentation of abdominal pain and hematuria. The most common etiologies for abdominal pain and hematuria are urinary tract infection or nephroliathiasis/urolithiasis. Laboratory values and imaging can often exclude these etiologies. The presence of hyperdense, circumferential mural thickening, either unilateral or bilateral, suggests suburothelial hemorrhage and should not be mistaken for a urothelial malignancy but instead lead to investigation for the presence of a bleeding diatheses. Although the process leads to luminal narrowing, only mild hydronephrosis has been rarely reported. Our case is unique in that forniceal rupture was present suggesting increase pressure in the renal collecting system. Although, at the time of imaging there was no hydronephrosis or hydroureter. The additional finding of retroperitoneal hemorrhage is a unique imaging triad (forniceal rupture, retroperitoneal hemorrhage and suburothelial hemorrhage) never before described in the literature. It is important for both clinicians and radiologists to be familiar with this diagnosis, and the spectrum of findings to ensure the appropriate diagnosis and treatment thereby eliminating unnecessary invasive treatment.
